# Supplementary material for: Sexual size dimorphism in mammals is associated with changes in the size of gene families related to brain development
Source: Nat Commun. 2024 Jul 24;15:6257. doi: 10.1038/s41467-024-50386-x (PMC11269740; doi:10.1038/s41467-024-50386-x)
Supplement: Supplementary file 1 — Suplemmentary information [file 41467_2024_50386_MOESM1_ESM.pdf]

Supplementary information for “Sexual size dimorphism in mammals is associated with changes in the size of gene families related to brain development”

Supplementary figures.

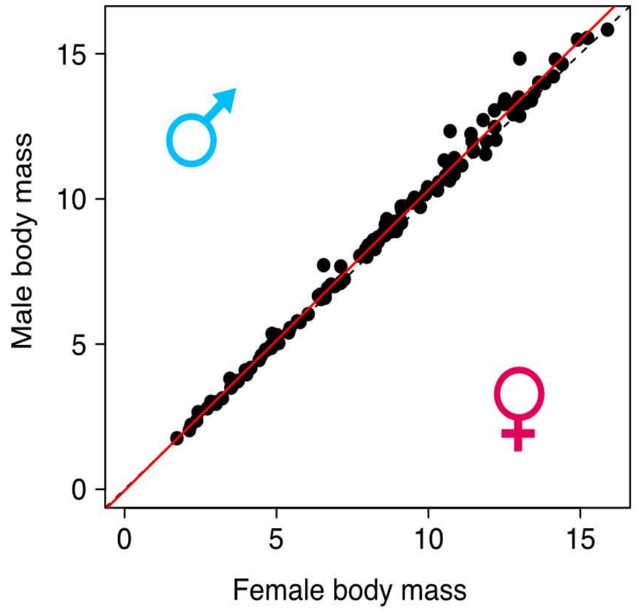

Supplementary figure 1. Test of Rensch’s rule. Asymmetry for sexual size dimorphism in mammals using logarithm transformed male and female body mass (kg). Red solid line: major-axis regression line; black dash line: slope = 1. The Rensch’s rule does apply in the set of 124 species examined in this study as larger male sizes are associated with increasing departure from one-to-one relation in size with females (slope = 1.034, upper – lower confidence intervals: 1.019 – 1.050, $p < 0.001$). Consistently, we found a significant and positive association between SSD and body mass ($r = 0.378$; $p < 0.001$).

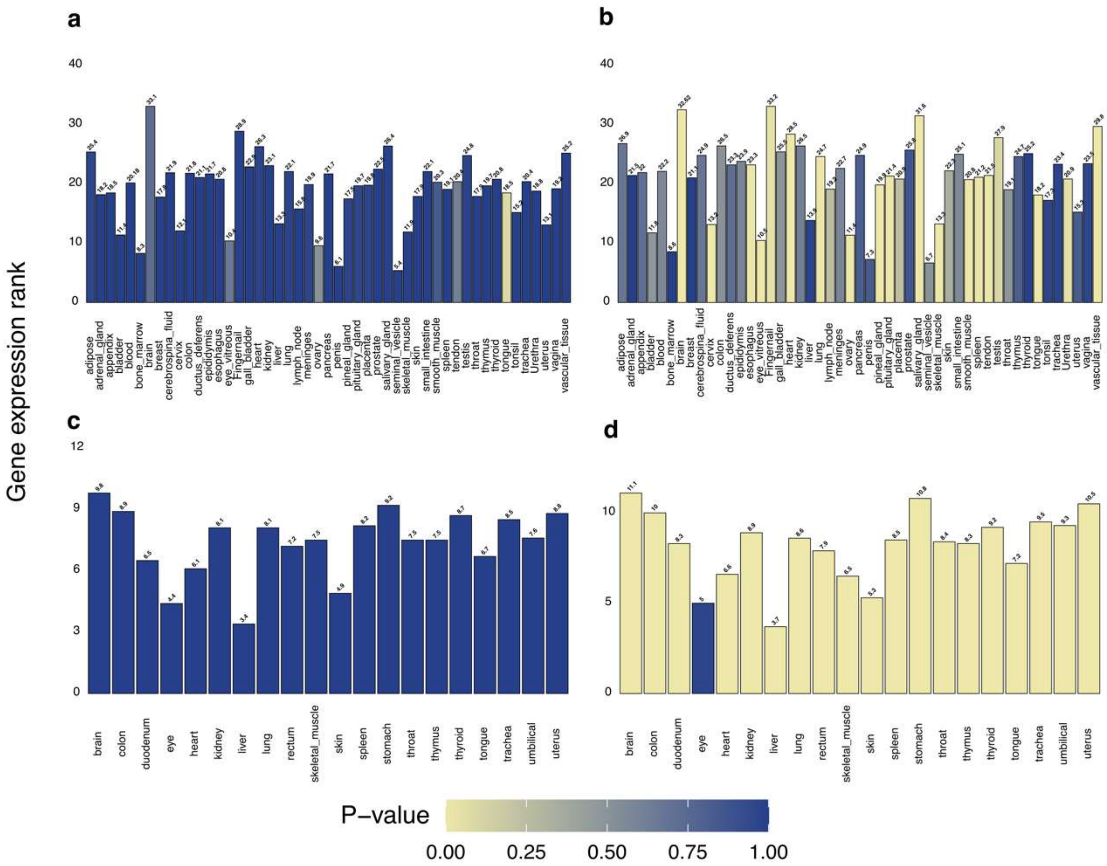

Supplementary figure 2. Tissue gene expression ranking in SSD-associated genes. The number on top of the bars represents the mean rank of the tissue. Lighter colours represent significant p-values. a) Expanding SSD-associated genes in adults, b) contracting SSD-associated genes in adults, c) expanding SSD-associated genes in prenatal stages and d) contracting SSD-associated genes in prenatal stages. Source data are provided as a source Data File.

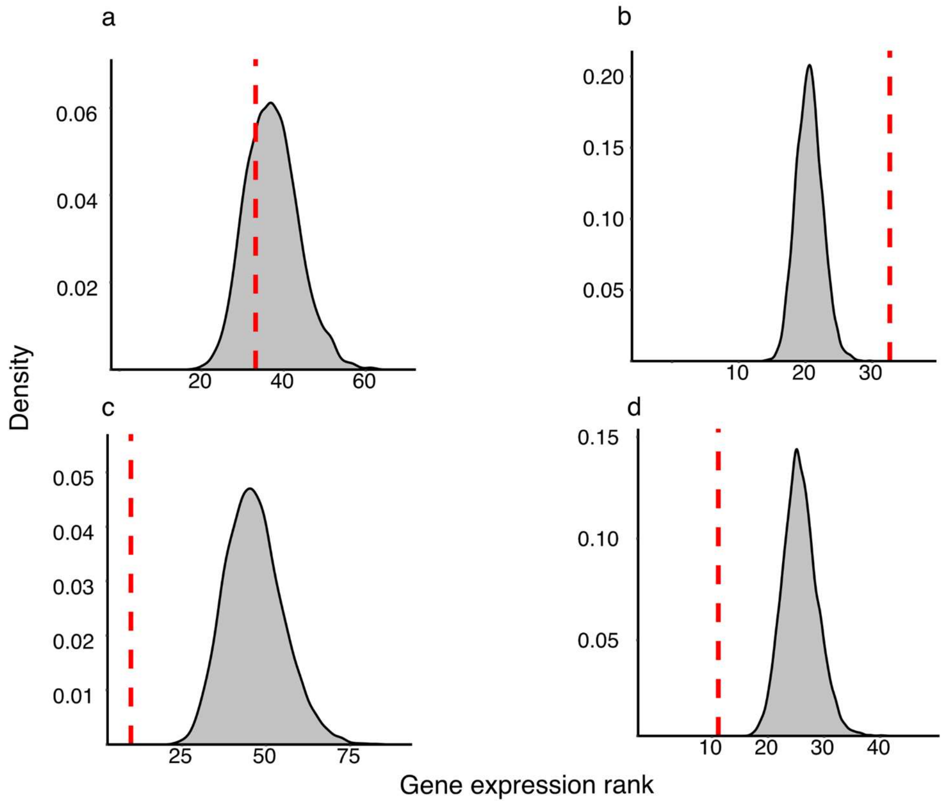

Supplementary figure 3. Brain gene expression rank in SSD-associated genes. The dashed red line shows the mean rank of the SSD-associated gene expression in the brain compared to other tissues. Meanwhile, the solid dark line represents the distribution of 10,000 bootstrapped ranks of SSD-associated genes in the brain. In adults, a) SSD-associated genes under expansion exhibit no statistically significant difference in average gene expression rank compared to bootstrapped results. We observe a significant increase for b) genes under contraction ($p < 0.001$). In prenatal stages, c) SSD-associated genes under expansion do not display a statistically significant increase but significantly lower ranks compared to bootstrapped results ($p < 0.001$). d) SSD-associated genes under contraction also demonstrate significantly lower expression ranks than bootstrapped expectations ($p < 0.01$).

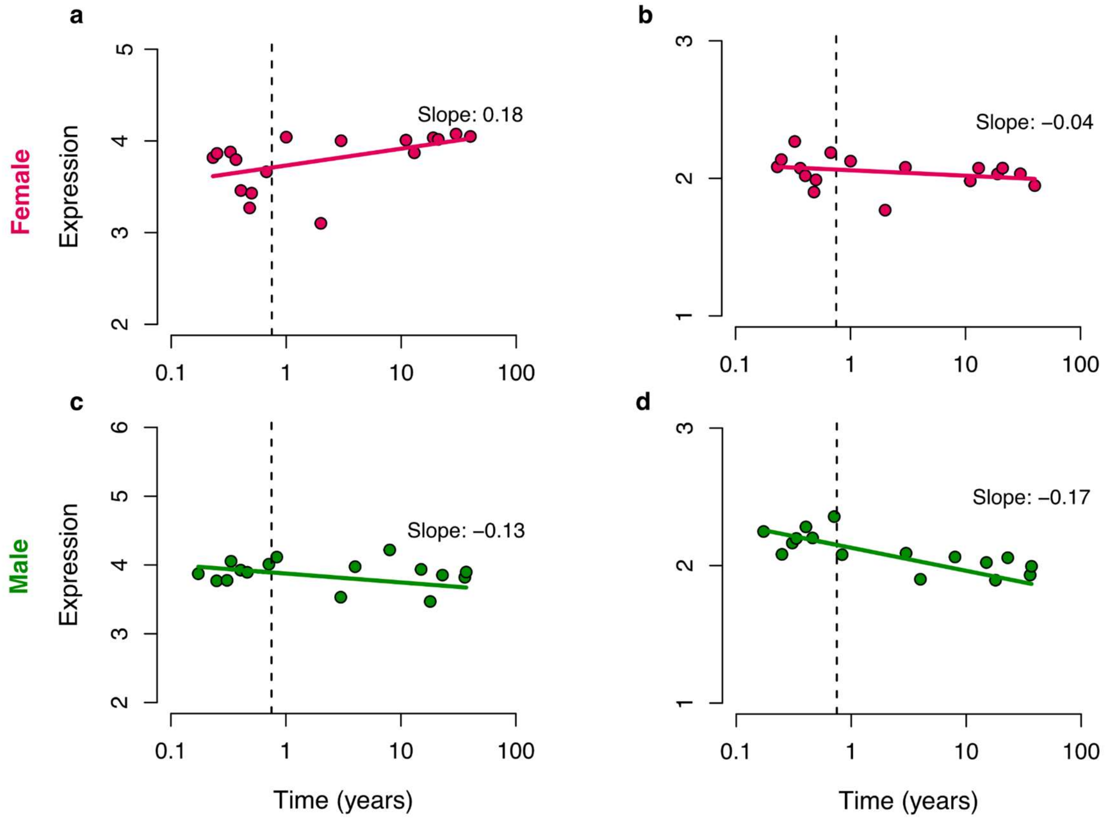

Supplementary figure 4. Temporal patterns of cortical expression of SSD-associated genes. Panels show average gene expression levels for A) SSD-associated under expansion genes in females, B) SSD-associated genes under contraction in females, C) SSD-associated genes under expansion in males and D) SSD-associated genes under contraction in males. The dashed line represents the end of gestation time (nine months). Source data are provided as a source Data File.

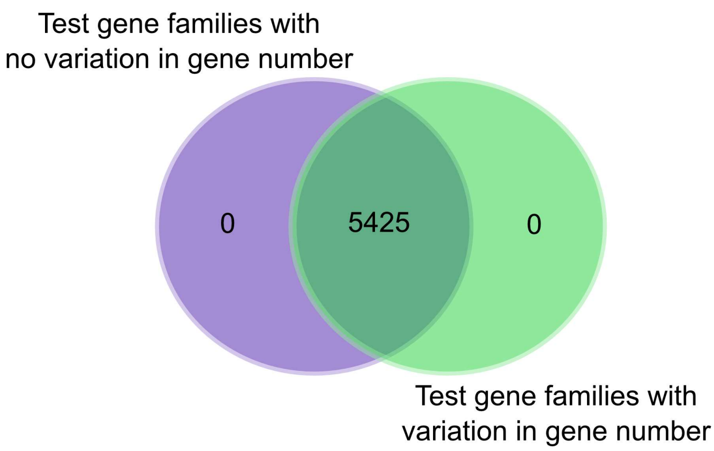

Supplementary figure 5. Gene families shared between PGLS with variation and no variation in gene numbers. The numbers in the diagram denote the total gene families that both PGLS share. Numbers in the outer regions of the diagram represent the gene families exclusive to each PGLS.

Supplementary Table 1. List of terms, abbreviations, and descriptions of brain structures used for sex-biased gene expression in temporal brain analysis.

Structure acronym	Structure name	Brain structure
**AMY**	amygdaloid complex	Cortex
**STC**	posterior (caudal) superior temporal cortex (area 22c)	Cortex
**CGE**	caudal ganglionic eminence	Cortex
**M1C**	primary motor cortex (area M1, area 4)	Cortex
**S1C**	primary somatosensory cortex (area S1, areas 3,1,2)	Cortex
**DTH**	dorsal thalamus	Cortex
**MD**	mediodorsal nucleus of thalamus	Cortex
**A1C**	primary auditory cortex (core)	Cortex
**DFC**	dorsolateral prefrontal cortex	Cortex
**STR**	striatum	Cortex
**V1C**	primary visual cortex (striate cortex, area V1/17)	Cortex
**OFC**	orbital frontal cortex	Cortex
**CB**	cerebellum	Cortex
**MFC**	anterior (rostral) cingulate (medial prefrontal) cortex	Cortex
**TCx**	temporal neocortex	Cortex
**MGE**	medial ganglionic eminence	Cortex
**HIP**	hippocampus (hippocampal formation)	Subcortex
**VFC**	ventrolateral prefrontal cortex	Subcortex
**ITC**	inferolateral temporal cortex (area TEv, area 20)	Subcortex
**URL**	upper (rostral) rhombic lip	Subcortex
**PCx**	parietal neocortex	Subcortex
**IPC**	posteroventral (inferior) parietal cortex	Subcortex
**Ocx**	occipital neocortex	Cerebellum
**CBC**	cerebellar cortex	Cerebellum
**M1C-S1C**	primary motor-sensory cortex (samples)	Cerebellum
