# Supplementary material for: Sexual size dimorphism in mammals is associated with changes in the size of gene families related to brain development
Source: Nat Commun. 2024 Jul 24;15:6257. doi: 10.1038/s41467-024-50386-x (PMC11269740; doi:10.1038/s41467-024-50386-x)
Supplement: Supplementary file 3 — Reporting Summary [file 41467_2024_50386_MOESM3_ESM.pdf]

nature portfolio

Corresponding author(s): Benjamin Padilla-Morales and Araxi O. Urrutia

Last updated by author(s): Mar 27, 2024

Reporting Summary

Nature Portfolio wishes to improve the reproducibility of the work that we publish. This form provides structure for consistency and transparency in reporting. For further information on Nature Portfolio policies, see our Editorial Policies and the Editorial Policy Checklist.

Statistics

For all statistical analyses, confirm that the following items are present in the figure legend, table legend, main text, or Methods section.

n/a	Confirmed	
☐	☒	The exact sample size (*n*) for each experimental group/condition, given as a discrete number and unit of measurement
☒	☐	A statement on whether measurements were taken from distinct samples or whether the same sample was measured repeatedly
☐	☒	The statistical test(s) used AND whether they are one- or two-sided<br>*Only common tests should be described solely by name; describe more complex techniques in the Methods section.*
☒	☐	A description of all covariates tested
☐	☒	A description of any assumptions or corrections, such as tests of normality and adjustment for multiple comparisons
☐	☒	A full description of the statistical parameters including central tendency (e.g. means) or other basic estimates (e.g. regression coefficient) AND variation (e.g. standard deviation) or associated estimates of uncertainty (e.g. confidence intervals)
☐	☒	For null hypothesis testing, the test statistic (e.g. *F*, *t*, *r*) with confidence intervals, effect sizes, degrees of freedom and *P* value noted<br>*Give P values as exact values whenever suitable.*
☒	☐	For Bayesian analysis, information on the choice of priors and Markov chain Monte Carlo settings
☒	☐	For hierarchical and complex designs, identification of the appropriate level for tests and full reporting of outcomes
☒	☐	Estimates of effect sizes (e.g. Cohen's *d*, Pearson's *r*), indicating how they were calculated

Our web collection on statistics for biologists contains articles on many of the points above.

Software and code

Policy information about availability of computer code

Data collection	To download all the used CDS sequences for this study from NCBI (16/08/20) we used the command wget (in a Linux machine) and the HTTPS link to each species CDS file in the NCBI FTP.
Data analysis	For Rensch rule we used the package dplyr (v. 1.0.10) and smatr (v. 3.4-8), to plot the results we used ggplot2 (v. 3.4.0)<br><br>We annotated gene families with Orthofinder version 2.4.0<br><br>We used R (version 4.2.2) and the packages logr (v. 1.3.3), tidyr (v. 1.2.1), ggpubr (v. 0.5.0) were used to perform genome completeness analyses. Then the packages ape (v. 5.6-2), caper (v. 1.0.1), nlme (v. 3.1-160) and parallel (4.2.2) were used to assess the PGLS for gene family expansion analysis. To plot the gene family expansion analysis the reshape (v. 0.8.9) and ggplot2 (v. 3.4.0) packages were used.<br><br>Gene Ontology enrichment analyses were assessed using the package parallel (v. 4.2.2), and to plot the heat map reshape (v. 0.8.9), lattice (v. 0.20-45) and RColorBrewer (v. 1.1-3)<br><br>For tissue expression analysis we used average rank analysis. logr (v. 1.3.3) was the only package used additional to basic R functions (version 4.2.2)<br><br>Temporal gene expression analyses were assessed using dplyr (v.1.0.10) and basic R functions (version 4.2.2) to calculate the average gene expression and average gene expression per gene.<br><br>Sex biased gene expression analyses were assessed by a fold change analysis using basic R functions (version 4.4.2) and the package logr (v.

1.3.3)

Scripts used in this study are available in the repository https://github.com/animazum/SSD-is-associated-with-brain-development-gene-family-sizes-in-mammals.git

For manuscripts utilizing custom algorithms or software that are central to the research but not yet described in published literature, software must be made available to editors and reviewers. We strongly encourage code deposition in a community repository (e.g. GitHub). See the Nature Portfolio guidelines for submitting code & software for further information.

Data

Policy information about availability of data

All manuscripts must include a data availability statement. This statement should provide the following information, where applicable:

- Accession codes, unique identifiers, or web links for publicly available datasets
- A description of any restrictions on data availability
- For clinical datasets or third party data, please ensure that the statement adheres to our policy

Phenotype data was retrieved from different online sources as described in supplementary table 1
All CDS sequences in this study were downloaded directly from Refseq NCBI FTP repository (16/08/20).
Mammalian phylogeny was retrieved from Timetree (16/08/20)
GO categories were downloaded from the Gene Ontology Consortium database (24/08/2022)
Human transcriptome data for 178 tissues (adults and prenatal) was downloaded from fantom5 project (05/10/2022)
Gene expression of protein coding genes for different brain areas was downloaded from Brainspan on November 2018
The data necessary to replicate this study is located in figshare database under the accession code: https://doi.org/10.6084/m9.figshare.22770731

Research involving human participants, their data, or biological material

Policy information about studies with human participants or human data. See also policy information about sex, gender (identity/presentation), and sexual orientation and race, ethnicity and racism.

Reporting on sex and gender	NA
Reporting on race, ethnicity, or other socially relevant groupings	NA
Population characteristics	NA
Recruitment	NA
Ethics oversight	NA

Note that full information on the approval of the study protocol must also be provided in the manuscript.

Field-specific reporting

Please select the one below that is the best fit for your research. If you are not sure, read the appropriate sections before making your selection.

☒ Life sciences ☐ Behavioural & social sciences ☐ Ecological, evolutionary & environmental sciences

For a reference copy of the document with all sections, see nature.com/documents/nr-reporting-summary-flat.pdf

Life sciences study design

All studies must disclose on these points even when the disclosure is negative.

Sample size	Sample size for CDS sequences, we used 146 sequences, each one coming from a different mammalian species. There were all the genomes available in refseq NCBI FTP in 16/08/20. Based on the phenotype data available, 124 species, we downloaded a phylogeny from time tree (16/08/20) with 124 species<br>For genome completion we filtered gene counts by single-copy genes (Removing the gene families with 0 or 1 genes per gene family), then we removed the gene families that does not have as many genes as the 80% of the number of species. Then we removed gene families that have 0 genes in 20% of the total number of species. Then we removed any gene family with variance 0. Finally, we remove the gene families that had a maximum of 3 genes in any species. The resulting output of this filtering was then used for the PGLS analysis.
Data exclusions	We excluded some species from analysis as we could not find quality data of their body mass for female and male individuals online.
Replication	Not applicable for this study, no experiments were performed.
Randomization	For PGLS we compared our experiment against 1000 radomized PGLS to assess if our findings were a generated by chance.<br>For tissue expression and gene expression analyses we compared our results against 1000 randomly selected samples

Blinding	Not applicable for this study, no experiments were performed.

Reporting for specific materials, systems and methods

We require information from authors about some types of materials, experimental systems and methods used in many studies. Here, indicate whether each material, system or method listed is relevant to your study. If you are not sure if a list item applies to your research, read the appropriate section before selecting a response.

Materials & experimental systems

n/a	Involved in the study
☒	☐ Antibodies
☒	☐ Eukaryotic cell lines
☒	☐ Palaeontology and archaeology
☒	☐ Animals and other organisms
☒	☐ Clinical data
☒	☐ Dual use research of concern
☒	☐ Plants

Methods

n/a	Involved in the study
☒	☐ ChIP-seq
☒	☐ Flow cytometry
☒	☐ MRI-based neuroimaging
