# Supplementary material for: Sexual size dimorphism in mammals is associated with changes in the size of gene families related to brain development
Source: Nat Commun. 2024 Jul 24;15:6257. doi: 10.1038/s41467-024-50386-x (PMC11269740; doi:10.1038/s41467-024-50386-x)
Supplement: Supplementary file 4 — Description of Additional Supplementary Files [file 41467_2024_50386_MOESM4_ESM.pdf]

Description of Additional Supplementary Files

File Name: Supplementary Data 1

Description: Output from gene family expansion/contraction analysis for sexual size dimorphism, along with their corresponding statistical significance per gene family.

File Name: Supplementary Data 2

Description: Average gene expression levels of brain areas and other tissues for adults and fetuses.

File Name: Supplementary Data 3

Description: Entrez Gene ID to GO category per gene with foldchange results and p-values.

File Name: Supplementary Data 4

Description: List of species used in the analysis with phenotypical data, including sources.

File Name: Supplementary Data 5

Description: List of sequences with respective species used for the orthology mapping analysis and links to ftp with download code.

File Name: Supplementary Data 6

Description: Output from gene family expansion/contraction analysis including zero variance gene families for sexual size dimorphism.

File Name: Supplementary Data 7

Description: List of species with encephalization index used in this research, along with references.
